# PF-06447475 Molecule Attenuates the Neuropathology of Familial Alzheimer’s and Coexistent Parkinson’s Disease Markers in PSEN1 I416T Dopaminergic-like Neurons

**DOI:** 10.3390/molecules30092034

**Published:** 2025-05-02

**Authors:** Diana Alejandra Quintero-Espinosa, Carlos Velez-Pardo, Marlene Jimenez-Del-Rio

**Affiliations:** Neuroscience Research Group, Institute of Medical Research, Faculty of Medicine, University Research Headquarters, Calle 62#52-59, Building 1, Laboratory 411/412, Medellin 050010, Colombia; dalejandra.quintero@udea.edu.co (D.A.Q.-E.); calberto.velez@udea.edu.co (C.V.-P.)

**Keywords:** apoptosis, Alzheimer’s disease, PF-06447475, presenilin, mutation, I416T, dopaminergic neurons, menstrual mesenchymal stromal cells

## Abstract

Familial Alzheimer’s disease (FAD) is a complex multifactorial disorder clinically characterized by cognitive impairment and memory loss. Pathologically, FAD is characterized by intracellular accumulation of the protein fragment Aβ42 (iAβ), hyperphosphorylated microtubule-associated protein TAU (p-TAU), and extensive degeneration of basal forebrain cholinergic neurons of the nucleus basalis of Meynert (NbM) and the medial septal nucleus (MSN), mainly caused by mutations in the amyloid precursor protein (*APP),* presenilin 1 (*PSEN1*), and *PSEN2* gene. Since the dopaminergic system may contribute to FAD symptoms, alterations in the nigro-hippocampal pathway may be associated with cognitive impairment in FAD. Interestingly, p-α-synuclein (p-α-Syn), Aβ, and p-TAU have been found to coexist in vulnerable regions of postmortem AD brains. However, the mechanism by which Aβ, p-TAU, and α-Syn coexist in DAergic neurons in AD brains has not been determined. We generated PSEN1 I416T dopaminergic-like neurons (DALNs) from I416T menstrual stromal cells (MenSCs) in *NeuroForsk 2.0* medium for 7 days and then cultured them in minimal culture medium (MCm) for another 4 days. On day 11, DALNs were analyzed for molecular and pathological markers by flow cytometry and fluorescence microscopy. We found that mutant DALNs showed increased accumulation of iAβ as well as increased phosphorylation of TAU at S202/T205 compared to WT DALNs. Thus, mutant DALNs exhibited typical pathological hallmarks of Alzheimer’s disease. Furthermore, PSEN1 I416T DALNs showed concomitant signs of OS as evidenced by the appearance of oxidized sensor protein DJ-1 (i.e., DJ-1C106-SO_3_) and apoptotic markers TP53, pS63-c-JUN, PUMA, and cleavage caspase 3 (CC3). Notably, these DALNs exhibited PD-associated proteins such as intracellular accumulation of α-Syn (detected as aggregates of pS129-α-Syn) and phosphorylation of LRRK2 kinase at residue S935. In addition, mutant DALNs showed a 17.16- and 6.17-fold decrease in DA-induced Ca^2+^ flux, compared to WT DALNs. These observations suggest that iAβ and p-TAU, together with p-α-Syn, and p-LRRK2 kinase, may damage DAergic neurons and thereby contribute to the exacerbation of neuropathologic processes in FAD. Remarkably, the LRRK2 inhibitor PF-06447475 (PF-475) significantly reversed PSEN1 I416T-induced neuropathological markers in DAergic neurons. PF-465 inhibitor reduced iAβ, oxDJ-1C106-SO_3_, and p-TAU. In addition, this inhibitor reduced pS935-LRRK2, pS129-αSYN, pS63-c-JUN, and CC3. We conclude that the observed neuroprotective effects of PF-475 are due to direct inhibition of LRRK2 activity and that the LRRK2 protein is upstream of the molecular cascade of apoptosis and proteinopathy. Our results suggest that PF-475 is an effective neuroprotective agent against endogenous PSEN1 I416T-induced neurotoxicity in DALNs coexisting with Parkinson’s disease markers. Therefore, PF-475 may be of great therapeutic value in FAD.

## 1. Introduction

Alzheimer’s disease (AD) is a complex, multifactorial, progressive, and chronic neurological disorder clinically characterized by cognitive impairment and memory loss [1] due to selective and extensive degeneration of basal forebrain cholinergic neurons in the nucleus basalis of Meynert (Ch4) and the medial septal nucleus (Ch1) [2]. Despite the generally accepted deterioration of the hippocampal and septal pathways [3], other pathways, such as the nigrostriatal pathway, have been reported to be impaired in AD patients [4,5]. Assuming that the dopaminergic system contributes to AD symptoms [6], alterations in the nigro-hippocampal pathway may be associated with cognitive impairment in AD. However, whether substantia nigral (SN) pathway impairment, i.e., the selective loss of dopaminergic (DAergic) neurons classically implicated in Parkinson’s disease (PD) [7], could influence AD-related symptoms (e.g., sensory and motor dysfunction) is not yet fully understood.

Alzheimer’s disease is biologically defined by the presence of amyloid-β plaques consisting mainly of the protein fragment Aβ42 and neurofibrillary tangles (NFTs) composed of hyperphosphorylated microtubule-associated protein TAU (p-TAU, Ref. [8]). Remarkably, the SN showed accumulation of Aβ plaques and NFTs in the postmortem brains of AD patients [9,10]. In addition, several reports have shown accumulation of the presynaptic protein α-synuclein (α-Syn), a neuropathologic marker associated with PD [11], in the SN of autopsy-confirmed AD brains [12]. These observations suggest that Aβ, p-TAU, and Lewy bodies (i.e., α-Syn) often coexist in AD brains [13], adding another layer of complexity to the pathophysiology of AD. Worst of all, the mechanism by which Aβ, p-TAU, and α-Syn coexist in DAergic neurons in AD brains has not yet been determined. Therefore, further investigation is needed to fully determine whether alterations in neuronal dopaminergic circuitry may be related to the clinical symptoms of AD. Unfortunately, there are no in vitro models to understand the interaction of Aβ, p-TAU, and α-Syn. In addition, it is still unknown whether other PD-associated proteins, such as leucine-rich repeat kinase 2 (LRRK2, Ref. [14]), might interact in AD. Therefore, studying the molecular basis of dopaminergic damage in reliable disease models may lead to the identification of targets in AD that could accelerate the discovery of disease-modifying therapies that delay the onset and slow the progression of AD.

Interestingly, familial AD (FAD) is clinically and biologically indistinguishable from sporadic AD (e.g., Refs. [15,16]), making FAD an attractive genetic model for the much more common AD. Indeed, FAD has an early onset (<60 years) and is caused by mutations in one or more of at least three genes known as amyloid precursor protein (APP), presenilin 1 (PSEN1), and presenilin 2 (PSEN2) (https://www.alzforum.org/mutations; accessed on 5 February 2025). Presenilin, a subunit of γ-secretase [17], is the aspartyl protease responsible for the generation of Aβ42 [18]. To date, more than 350 different PSEN1 mutations have been reported to cause FAD (https://www.alzforum.org/mutations/psen-1, accessed 10 March 2025). Among them, PSEN1 I416T (p.I416T), resulting from an isoleucine-to-threonine substitution, causes familial early-onset AD (EOAD) in a large Colombian family [19]. Unfortunately, there are no effective therapies available. Therefore, innovative therapeutic approaches are currently needed. Recently, our research group recapitulated the neuropathology of FAD in PSEN1 I416T cholinergic-like neurons (ChLNs) derived from menstrual stromal cells (MenSCs, Ref. [20]). Indeed, mutant but not wild-type (WT) PSEN1 ChLNs showed accumulation of intracellular Aβ (iAβ) fragments, tau phosphorylation (p-S202/T205 TAU), extracellular Aβ42 (eAβ42), oxidative stress (OS), loss of mitochondrial membrane potential (ΔΨm), DNA fragmentation, activation of apoptogenic proteins (TP53, PUMA, c-Jun), and Ca^2+^ influx dysregulation in response to acetylcholine (ACh) stimuli. However, whether MenSCs-derived dopamine-like neurons (DALNs) reproduce the neuropathological markers associated with FAD PSEN 1 I416T and PD (e.g., phosphorylation of LRRK2, accumulation of p-α-Syn) remains to be determined. Notably, the multifunctional kinase LRRK2 [21,22] was recently shown to be endogenously phosphorylated at residue S935 in PSEN1 E280A ChLNs [23]. Furthermore, phosphorylated p-αSyn at pathogenic residue S129 (pS129-αSyn) was also found to co-localize with pS935-LRRK2 in the same primary mutant ChLNs [23]. Since pS935-LRRK2 not only phosphorylates pS129-αSyn [24], but may also be involved in the regulation of OS [25,26] and apoptosis [27,28,29], among other cellular functions [30,31], in typical PD cholinergic neurons, we speculate that the neuropathological hallmark of PD α-Syn may coexist with AD and that the LRRK2 protein may function in both diseases. However, the mechanism by which LRRK2 induces the coexistence of α-Syn, Aβ, and TAU in PSEN1 mutant DALNs is unknown [32]. Therefore, elucidating the role of LRRK2 may provide important information for understanding the pathogenesis of FAD and refining future clinical trials. Given that inhibition of LRRK2 kinase protects nerve-like differentiated cells from OS-induced cell death [33] and from PD-associated environmental toxicants [34], we theorized that specific inhibition of LRRK2 might protect PSEN1 mutant DALNs from Aβ-induced OS and apoptosis.

The aim of the present investigation was to determine whether PSEN1 I416T MenSCs differentiate into DALNs when cultured in NeuroForsk 2.0 medium [35], a modified version of the previous NeuroForsk medium originally developed in our laboratory [36], reproduce the typical features of FAD as previously shown in the mutant ChLNs [20], and whether DALNs express neuropathologic markers of PD such as α-Syn, which is associated with phosphorylation of LRRK2 kinase. We found that, similar to PSEN1 I416T MenSCs-derived ChLNs, PSEN1 I416T MenSCs can transdifferentiate into PSEN1 I416T DALNs by growing in NeuroForsk 2.0 medium for 7 days and then being cultured in minimal culture medium (MCm) for another 4 days. Compared to WT PSEN1 DALNs, PSEN1 I416T DALNs showed signs of increased accumulation of iAβ and increased phosphorylation of TAU at S202/T205. Thus, PSEN1 I416T DALNs molecularly mimic FAD. Furthermore, PSEN1 I416T DALNs showed signs of OS as evidenced by the appearance of DJ-1C106-SO3 and apoptotic markers such as TP53, PUMA, pS63-c-JUN, and cleavage caspase 3 (CC3) at day 11 of transdifferentiation. Mutant DALNs also exhibited DA-induced Ca^2+^ flux dysregulation compared to control (WT) DALNs. In addition, PSEN1 I416T DALNs displayed PD-associated proteins such as intracellular accumulation of α-Syn (detected as aggregates of pS129-α-Syn) and phosphorylation of LRRK2 at residue S935. Notably, exposure of cells to the potent LRRK2 inhibitor PF-06447475 (PF-475, Ref. [37]) decreased iAβ and α-Syn accumulation, reduced OS, decreased expression of apoptotic markers, blocked p-TAU, reversed LRRK2 kinases, and restored Ca^2+^ influx in PSEN1 I416T DALNs. Our results suggest that PF-475 is an effective neuroprotective agent against endogenously generated pathological iAβ, OS, α-Syn, p-LRRK2, p-TAU, and apoptosis in PSEN1-derived DALNs. These observations suggest that iAβ and p-TAU, together with α-Syn and LRRK2 kinase, may damage DAergic neurons and thereby contribute to the exacerbation of the neuropathologic processes of FAD. Our results suggest that PF-475 is an effective neuroprotective agent against endogenous PSEN1 I416T-induced neuropathology in DALNs. Therefore, PF-475 may be of great therapeutic value in FAD.

## 2. Results

### 2.1. PSEN1 I416T Menstrual Stromal Cells (MenSCs) Transdifferentiated into Dopaminergic-like Neurons (DALNs)

**Figure 1:**
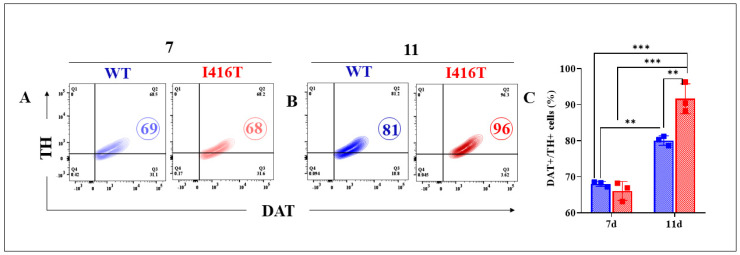

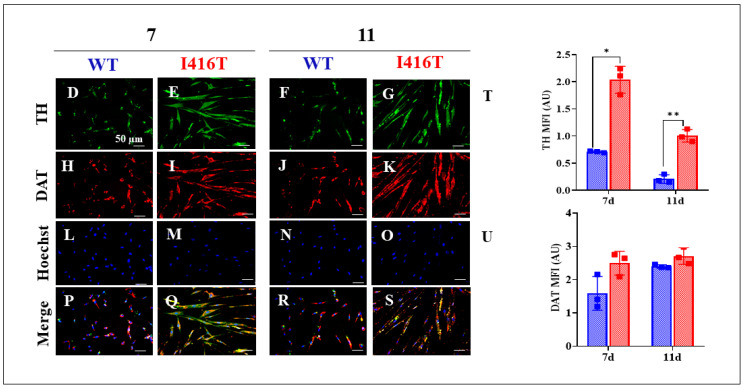
PSEN1 I416T MenSCs transdifferentiated into dopaminergic-like neurons (DALNs) express dopaminergic markers Tyrosine hydroxylase (TH) and dopamine transporter (DAT). Representative density plot figures showing TH/DAT double-positive population in WT PSEN1 and PSEN1 I416T DALNs after (**A**) 7 and (**B**) 11 days of transdifferentiation. (**C**) Percentage of TH/DAT double-positive population. (**D**–**S**) Representative fluorescence microscopy photographs showing dopaminergic markers DAT (red fluorescence) and TH (green fluorescence). The nuclei were stained with Hoechst 33,342 (blue). (**T**,**U**) Mean fluorescence intensity (MFI) quantification of images of DAT (red fluorescence) and TH (green fluorescence). The figures represent 1 out of 3 independent experiments. One-way ANOVA followed by Tukey’s test (** *p* < 0.002 (**C**), *** *p* < 0.001 (**C**)) or post hoc test Bonferroni (* *p* < 0.033 (**T**), ** *p* < 0.002 (**T**)). The data are presented as the mean ± SD of three independent experiments (*n* = 3). Bars in blue represent WT cells. Bars in red represent mutant cells. Image magnification, 200×.

We first investigated whether MenSCs are capable of transdifferentiating into DALNs. As shown in Figure 1, both WT and mutant cells expressed the typical dopaminergic lineage markers tyrosine hydroxylase (TH) and dopamine transporter (DAT) by 69%, according to flow cytometry analysis (Figure 1A,C). As these cells stained for the double marker TH/DAT, which are involved in dopamine synthesis [38] and transport [39], the cells could be classified as dopaminergic-like neurons (DALNs) in nature. To further evaluate whether a change in culture conditions affects such a transdifferentiation process, DALNs cultured on day 7 were exposed to minimal culture medium (MCm) containing fetal bovine serum (1% FBS) and low-glucose DMEM for an additional 4 days. Figure 1B shows that both WT and mutant DALNs significantly expressed 81% and 96% TH/DAT-positive cells, respectively; however, mutant DALNs expressed more TH/DAT than WT DAergic neurons (Figure 1C). Similar results were obtained by fluorescence microscopy analysis (Figure 1D–U). 

### 2.2. PSEN1 I416T Induces Aggregation of Intracellular Aβ (iAβ), Oxidative Stress (OS), and Abnormal Phosphorylation of TAU in Dopaminergic-like Neurons (DALNs)

**Figure 2:**
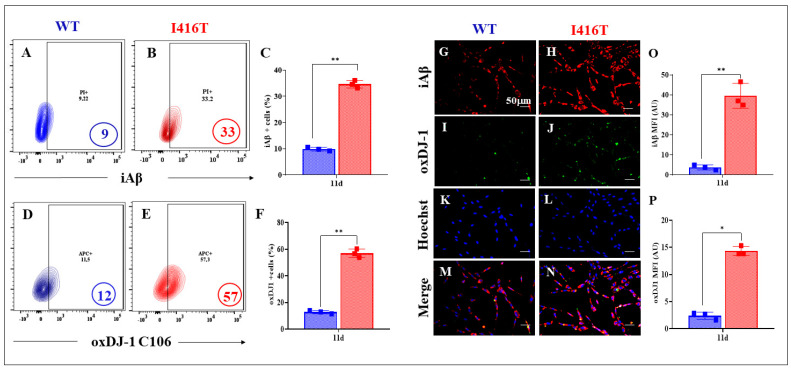
PSEN1 I416T DALNs express abundant iAβ and shows oxidation of DJ-1 after 11 days of transdifferentiation. Representative flow cytometry contour plots show intracellular amyloid-beta (iAβ) in (**A**) WT PSEN1 and (**B**) PSEN1 I416T DALNs. (**C**) Percentage of intracellular amyloid-beta (iAβ). Representative flow cytometry contour plots show oxidized protein DJ-1 (DJ-1 C106-SO_3_) in (**D**) WT PSEN1 and (**E**) PSEN1 I416T DALNs. (**F**) Percentage of oxDJ-1 (DJ-1 C106-SO_3_). WT PSEN1 and PSEN1 I416T DALNs were stained with primary antibodies against iAβ (**G**,**H**), oxDJ-1 (**I**,**J**), Hoechst (**K**,**L**), and merged (**M**,**N**). Positive red fluorescence reflects the cytoplasmic presence of iAβ protein, positive green fluorescence reflects the cytoplasmic presence of oxDJ-1, and positive blue fluorescence reflects nuclei. Quantification of the (**O**) iAβ and (**P**) oxDJ-1 mean fluorescence intensity (MFI). The figures represent one out of three independent experiments. One-way ANOVA followed by Tukey’s test. The data are expressed as the mean ± SD of three independent experiments (n = 3); * *p* < 0.033 (**P**), ** *p* < 0.01. (**C**,**F**), ** *p* < 0.002 (**O**). Bars in blue represent WT cells. Bars in red represent mutant cells. Image magnification, 200×.

Next, we wanted to evaluate whether PSEN1 I416T DALNs show the typical neuropathologic markers of AD at a similar time point (11 days). Flow cytometry analysis reveals that WT DALNs accumulated a basal amount of iAβ (9%, Figure 2A,C), whereas mutant DALNs accumulated a high percentage of intrinsic iAβ (33%, Figure 2B,C), i.e., mutant cells increased iAβ by +267% compared to WT cells. Oxidative stress, as evidenced by the oxidation of the stress sensor protein DJ-1 to DJ-1C106SO_3_, was much more pronounced in the mutant than in the WT. Indeed, PSEN1 I416T DALNs increased DJ-1C106SO_3_ by +375% compared to WT cells (Figure 2D–F). Immunofluorescence microscopy analysis confirmed the above observations (Figure 2G–P).

**Figure 3:**
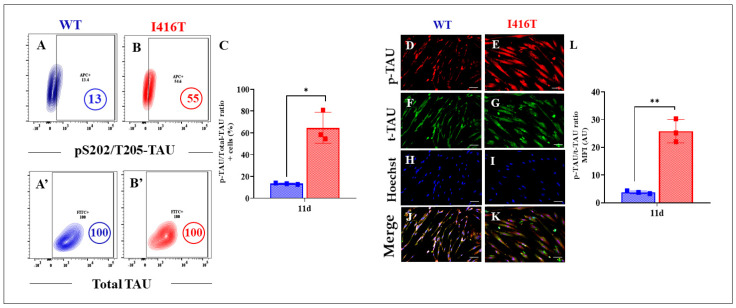
PSEN1 I416T DALNs shows high levels of phosphorylated protein TAU (pS202/T205-TAU) after 11 days of transdifferentiation. Representative flow cytometry contour plots show intracellular pS202/T205-TAU in (**A**) WT PSEN1 and (**B**) PSEN1 I416T DALNs. Representative flow cytometry contour plots show total TAU protein (t-TAU) in (**A’**) WT PSEN1 and (**B’**) PSEN1 I416T DALNs. (**C**) Percentage of p-TAU/t-TAU ratio. WT PSEN1 and PSEN1 I416T DALNs were stained with primary antibodies against pS202/T205-TAU (**D**,**E**), t-TAU (**F**,**G**), Hoechst (**H**,**I**), and merged (**J**,**K**). Positive red fluorescence reflects the cytoplasmic presence of pS202/T205-TAU protein, positive green fluorescence reflects the cytoplasmic presence of t-TAU, and positive blue fluorescence reflects nuclei. (**L**) Quantification of the pS202/T205-TAU/t-TAU ratio mean fluorescence intensity (MFI). The figures represent one out of three independent experiments. One-way ANOVA followed by Tukey’s test. The data are presented as the mean ± SD of three independent experiments (n = 3); * *p* < 0.05, ** *p* < 0.002. Bars in blue represent WT cells. Bars in red represent mutant cells. Image magnification, 200×.

Similar to iAβ and oxDJ-1, PSEN1 I416T DALNs increased the phosphorylation of TAU at residue S202/T205 by +323% compared to WT DALNs (Figure 3A–C). Immunofluorescence microscopy analysis reveals similar results (Figure 3D–L).

### 2.3. PSEN1 I416T Induces Phosphorylation of LRRK2 and α-Synuclein in Dopaminergic-like Neurons (DALNs)

**Figure 4:**
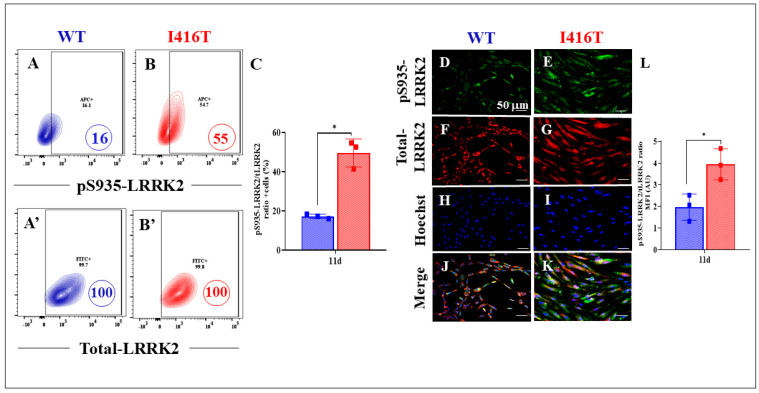
PSEN1 I416T DALNs increase levels of phosphorylated protein LRRK2 (pS935-LRRK2) after 11 days of transdifferentiation. Representative flow cytometry contour plots show intracellular pS935-LRRK2 in (**A**) WT PSEN1 and (**B**) PSEN1 I416T DALNs. Representative flow cytometry contour plots show total LRRK2 protein (t-LRRK2) in (**A’**) WT PSEN1 and (**B’**) PSEN1 I416T DALNs. (**C**) Percentage of pS935-LRRK2/t-LRRK2 ratio. WT PSEN1 and PSEN1 I416T DALNs were stained with primary antibodies against pS935-LRRK2 (**D**,**E**), t-LRRK2 (**F**,**G**), Hoechst (**H**,**I**), and merged (**J**,**K**). Positive green fluorescence reflects the cytoplasmic presence of pS935-LRRK2 protein, positive red fluorescence reflects the cytoplasmic presence of t-LRRK2, and positive blue fluorescence reflects nuclei. (**L**) Quantification of the pS935-LRRK2/t-LRRK2 ratio mean fluorescence intensity (MFI). The figures represent one out of three independent experiments. One-way ANOVA followed by Tukey’s test. The data are presented as the mean ± SD of three independent experiments (n = 3); * *p* < 0.033. Bars in blue represent WT cells. Bars in red represent mutant cells. Image magnification, 200×.

**Figure 5:**
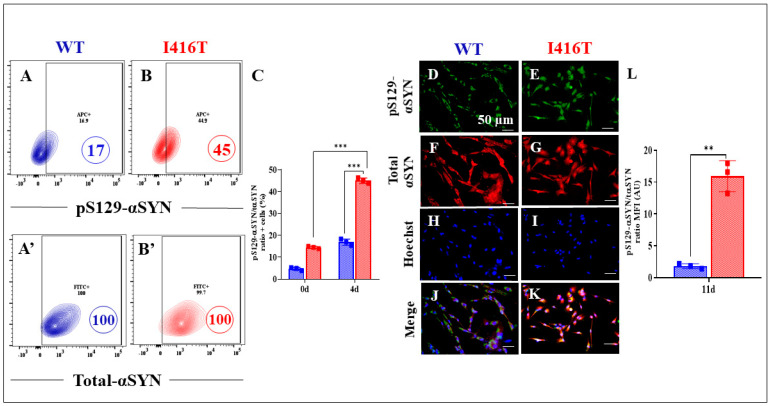
PSEN1 I416T DALNs increase levels of phosphorylated protein alpha synuclein (pS129-αSYN) after 11 days of transdifferentiation. Representative flow cytometry contour plots show intracellular pS129-αSYN in (**A**) WT PSEN1 and (**B**) PSEN1 I416T DALNs. Representative flow cytometry contour plots show total alpha synuclein protein (t-αSYN) in (**A’**) WT PSEN1 and (**B’**) PSEN1 I416T DALNs. (**C**) Percentage of pS129-αSYN/t-αSYN ratio. WT PSEN1 and PSEN1 I416T DALNs were stained with primary antibodies against pS129-αSYN (**D**,**E**), t-αSYN (**F**,**G**), Hoechst (**H**,**I**), and merged (**J**,**K**). Positive green fluorescence reflects the cytoplasmic presence of pS129-αSYN, positive red fluorescence reflects the cytoplasmic presence of t-αSYN protein, and positive blue fluorescence reflects nuclei. (**L**) Quantification of the pS129-αSYN/t-αSYN ratio mean fluorescence intensity (MFI). The figures represent one out of three independent experiments. One-way ANOVA followed by Tukey’s test. The data are presented as the mean ± SD of three independent experiments (n = 3); ** *p* < 0.002, *** *p* < 0.001. Bars in blue represent WT cells. Bars in red represent mutant cells. Image magnification, 200×.

In addition, we examined whether PSEN1 I146T DALNs have abnormal p-LRRK2 and p-α-Syn. Indeed, compared with WT DALNs (Figure 4A and Figure 5A), PSEN1 I146T DALNs show increases in pS935-LRRK2 (Figure 4B) and pS129-α-Syn (Figure 5B) of +244% (Figure 4C) and +165% (Figure 5C), respectively. Immunofluorescence microscopy analysis shows similar results (Figure 4D–L and Figure 5D–L).

### 2.4. PSEN1 I416T Dopaminergic-like Neurons (DALNs) Show Signs of Apoptosis

**Figure 6:**
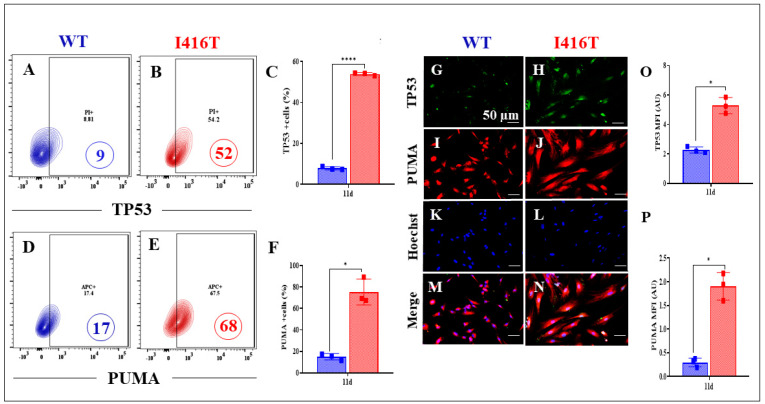
PSEN1 I416T DALNs increase levels of TP53 and P53 upregulated modulator of apoptosis (PUMA) after 11 days of transdifferentiation. Representative flow cytometry contour plots show TP53 protein level in (**A**) WT PSEN1 and (**B**) PSEN1 I416T DALNs. (**C**) Percentage of TP53 expression. Representative flow cytometry contour plots show P53 upregulated modulator of apoptosis (PUMA) protein in (**D**) WT PSEN1 and (**E**) PSEN1 I416T DALNs. (**F**) Percentage of PUMA. In addition, WT PSEN1 and PSEN1 I416T DALNs were stained with primary antibodies against TP53 (**G**,**H**), PUMA (**I**,**J**), Hoechst (**K**,**L**), and merged (**M**,**N**). Positive green fluorescence reflects the cytoplasmic presence of TP53 protein, positive red fluorescence reflects the cytoplasmic presence of PUMA, and positive blue fluorescence reflects nuclei. Quantification of the of (**O**) TP53 and (**P**) PUMA mean fluorescence intensity (MFI) in WT PSEN1 and PSEN1 I416T DALNs. The figures represent one out of three independent experiments. The data are presented as the mean ± SD of three independent experiments (n = 3). One-way ANOVA followed by Tukey’s test. The data are expressed as the mean ± SD; * *p* < 0.033, **** *p* < 0.0001. Bars in blue represent WT cells. Bars in red represent mutant cells. Image magnification, 200×.

**Figure 7:**
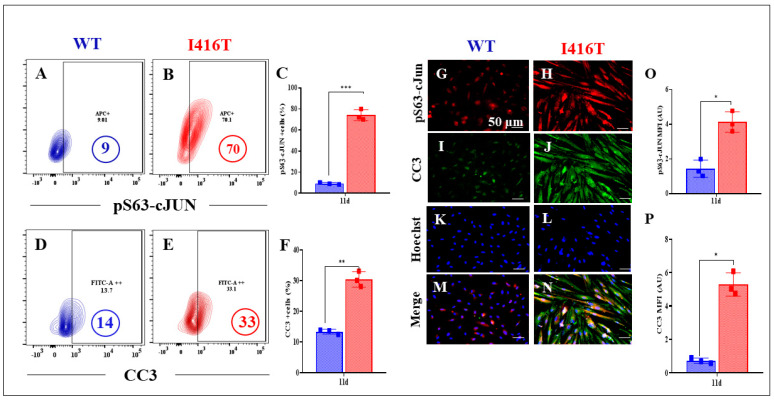
PSEN1 I416T DALNs increase levels of c-JUN phosphorylated at serine 63 (pS63-c-JUN) and cleaved caspase-3 (CC3) after 11 days of transdifferentiation. Representative flow cytometry contour plots show pS63-c-JUN protein level in (**A**) WT PSEN1 and (**B**) PSEN1 I416T DALNs. (**C**) Percentage of pS63-c-JUN expression. Representative flow cytometry contour plots show cleaved caspase-3 (CC3) protein in (**D**) WT PSEN1 and (**E**) PSEN1 I416T DALNs. (**F**) Percentage of CC3. WT PSEN1 and PSEN1 I416T DALNs were stained with primary antibodies against pS63-c-JUN (**G**,**H**), CC3 (**I**,**J**), Hoechst (**K**,**L**), and merged (**M**,**N**). Positive red fluorescence reflects the cytoplasmic presence of pS63-c-JUN, positive green fluorescence reflects the cytoplasmic presence of CC3, and positive blue fluorescence reflects nuclei. Quantification of the of (**O**) pS63-c-JUN and (**P**) CC3 mean fluorescence intensity (MFI). The figures represent one out of three independent experiments. One-way ANOVA followed by Tukey’s test. The data are presented as the mean ± SD of three independent experiments (n = 3); * *p* < 0.033. ** *p* < 0.02, *** *p* < 0.0002. Bars in blue represent WT cells. Bars in red represent mutant cells. Image magnification, 200×.

DALNs were then examined for the presence of apoptotic signals. In contrast to WT DALNs (Figure 6A,D and Figure 7A,D), PSEN1 DALNs show an abnormal upregulation of the pro-apoptotic transcription factor TP53 (Figure 6B), the Bcl-2-related BH-3-only protein PUMA (Figure 6E), the apoptogenic transcription factor pS63-c-JUN (Figure 7B), and cleaved caspase 3 (CC3, Figure 7E) by +478% (Figure 6C), 300% (Figure 6F), 678% (Figure 7C), and 136% (Figure 7F), respectively. Similar results were obtained by immunofluorescence microscopy analysis (Figure 6G–P and Figure 7G–P).

### 2.5. PSEN1 I416T Dopaminergic-like Neurons (DALNs) Are Irresponsive to Dopamine-Induced Transient Ca^2+^ Influx

**Figure 8:**
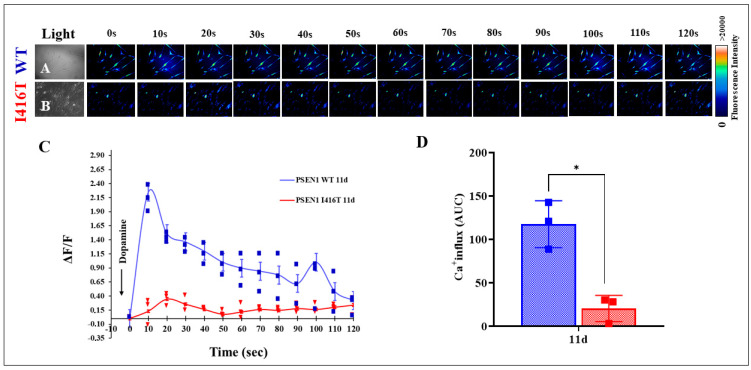
PSEN1 I416T DALNs exhibit dysfunctional Ca^2+^ influx response to dopamine (DA) at day 11 of transdifferentiation. Representative time-lapse images (0, 10, 20, 30, 40, 60, 70, 80, 90, 100, 110, 120 s) of Ca^2+^ fluorescence in response to DA (arrow) in (**A**) WT PSEN1 and (**B**) PSEN1 I416T DALNs. Color contrast indicates fluorescence intensity: dark blue < light blue < green < red. (**C**) Graph showing ΔF/F and (**D**) area under the curve (AUC). The figures represent 1 out of 3 independent experiments. One-way ANOVA followed by Tukey’s test. The data are presented as the mean ± SD of three independent experiments (n = 3); * *p* < 0.033,. Bars in blue represent WT cells. Bars in red represent mutant cells. Image magnification, 200×.

We further investigated whether WT and PSEN1 I416T DALNs responded to dopamine (DA) stimuli. As shown in Figure 8, DA induced a transient increase in intracellular Ca^2+^ in WT PSEN1 DALNs at day 11 of transdifferentiation (Figure 8A,C). The mean fluorescence change (ΔF/F) was 2.248 ± 0.03 with a mean duration of 10 s (n = 20 DALN cells imaged, N = 3 dishes) and 1.00 ± 0.05 with a mean duration of 100 s (n = 20 DALN cells imaged, N = 3 dishes), corresponding to cytoplasmic Ca^2+^ responses to Fluo-3-mediated imaging. PSEN1 I416T DALNs show significantly lower intracellular Ca^2+^ in response to DA treatment (Figure 8B,C). The mean fluorescence change (ΔF/F) was 0.131 ± 0.03 with a mean duration of 10 s (n = 20 DALN cells imaged, N = 3 dishes) and 0.162 ± 0.02 with a mean duration of 100 s (n = 20 DALN cells imaged, N = 3 dishes). Nevertheless, we found a significant difference in the ΔF/F response in PSEN1 I416T DALNs exposed to DA compared to that in WT DALNs (Figure 8C,D). In fact, WT DALNs responded more strongly to DA, i.e., a 17.16-fold increase at 10 s (fast response to DA) and a 6.17-fold increase at 100 s (slow response to DA) in DA-induced transient Ca^2+^ influx compared to PSEN1 I416T DALNs (Figure 8C,D).

### 2.6. LRRK2 Inhibitor PF-06447475 (PF-475) Attenuates the Neuropathologic Markers iAβ, oxDJ-1, and p-TAU Associated with FAD in PSEN1 I416T Dopaminergic-like Neurons (DALNs)

**Figure 9:**
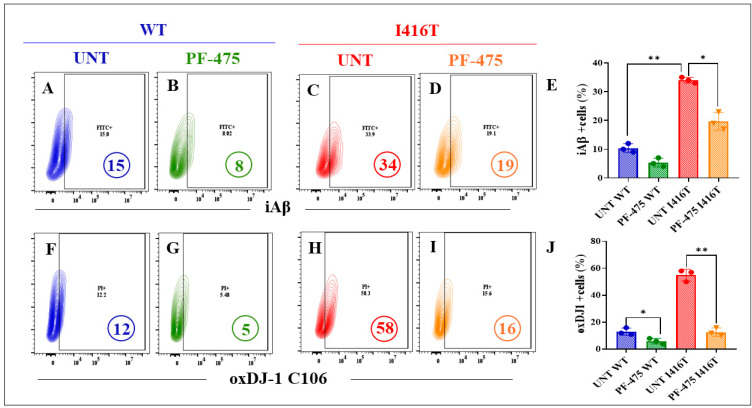
The LRRK2 inhibitor PF-06447475 (PF-475) blunts the expression of iAβ and oxidation of DJ-1 in PSEN1 I416T DALNs. Representative flow cytometry contour plots show intracellular amyloid-beta (iAβ) in (**A**,**C**) untreated or (**B**,**D**) treated cells with PF-06447475 LRRK2 inhibitor (PF-475). (**E**) Percentage of intracellular amyloid-beta (iAβ). (**F**–**I**) Representative flow cytometry contour plots show oxidized protein DJ-1 (DJ-1 C106-SO_3_). (**J**) Percentage of oxDJ-1 (DJ-1 C106-SO_3_). One-way ANOVA followed by Tukey’s test. The data are presented as the mean ± SD of three independent experiments (n = 3); * *p* < 0.033, ** *p* < 0.002.

**Figure 10:**
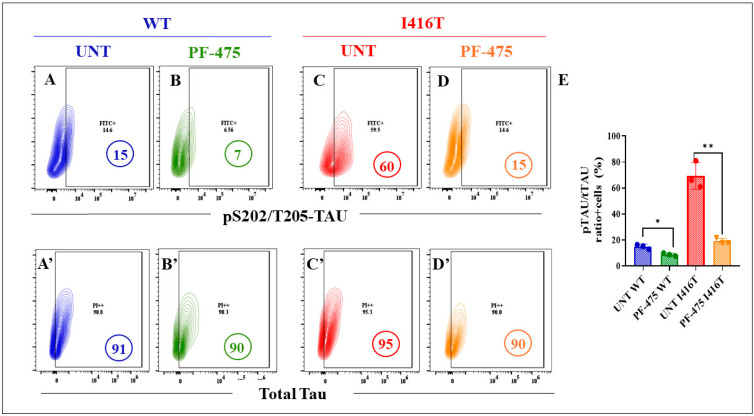
The LRRK2 inhibitor PF-06447475 reduces the levels of phosphorylated protein TAU (pS202/T205-TAU) in PSEN1 I416T DALNs. Representative flow cytometry contour plots show intracellular pS202/T205-TAU in (**A**,**C**) untreated or (**B**,**D**) treated cells with PF-475. Representative flow cytometry contour plots show total-TAU protein in (**A’**,**C’**) untreated or (**B’**–**D’**) treated cells with PF-475. (**E**) Percentage of p-TAU/t-TAU ratio in untreated or treated cells with PF-475. One-way ANOVA followed by Tukey’s test. The data are presented as the mean ± SD of three independent experiments (n = 3); * *p* < 0.033, and ** *p* < 0.002.

The above observations prompted us to evaluate the potential of the LRRK2 inhibitor PF-475 to reverse the PSEN1 I416T-induced pathogenic phenotype in DALNs. Since DALNs (DALNs and likely cells primed to transdifferentiate into dopaminergic neurons, Figure 1A–C) are phenotypically dopaminergic by day 7, WT and mutant cells were left untreated or treated with PF-475 from day 7 to day 11. The markers iAβ, oxDJ-1, and p-TAU were then evaluated by flow cytometry. The results show that the basal levels of accumulated iAβ (Figure 9A) were reduced by −47% in WT DALNs when exposed to PF-475 (Figure 9B,E), and the abnormal accumulation of iAβ (Figure 9C) was reduced by −44% in mutant DALNs when treated with PF-475 (Figure 9D,E). Analysis of oxD-1 shows a similar trend to iAβ. Basal levels of DJ-1 C106SO_3_ in WT DALNs (Figure 9F) and high levels of oxDJ-1 in mutant cells (Figure 9H) were reduced by −58% (Figure 9G) and −72% (Figure 9I), respectively, when WT and mutant DALNs were exposed to PF-475 (Figure 9J). Notably, PF-475 not only reduced basal p-TAU (Figure 10A) by −50% in WT cells (Figure 10B) but also significantly reduced the highly phosphorylated protein TAU at S202/T2025 (Figure 10C) by −73% (Figure 10D) in PSEN1 I416T DALNs (Figure 10E).

### 2.7. LRRK2 Inhibitor PF-06447475 (PF-475) Reduces the Neuropathologic p-LRRK2 Kinase and p-α-Syn Associated with PD in PSEN1 I416T Dopaminergic-like Neurons (DALNs)

**Figure 11:**
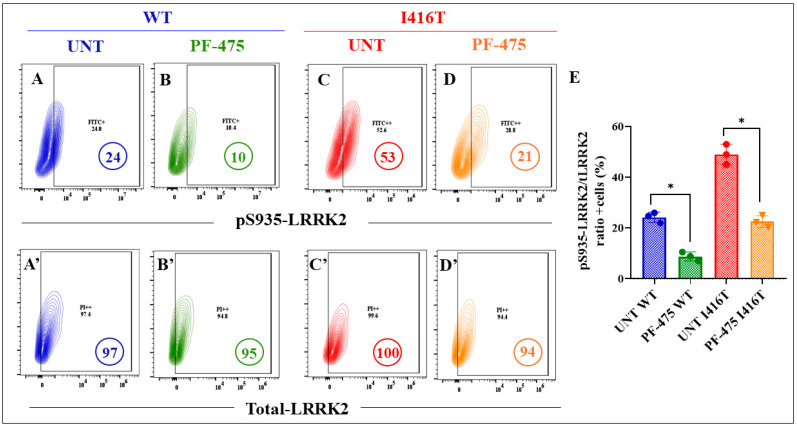
The LRRK2 inhibitor PF-06447475 reduces the levels of phosphorylated protein LRRK2 (pS935-LRRK2) in PSEN1 I416T DALNs. Representative flow cytometry contour plots show intracellular pS935-LRRK2 in (**A**,**C**) untreated or (**B**,**D**) treated cells with PF-475. Representative flow cytometry contour plots show total LRRK2 protein (**A’**,**C’**) untreated or (**B’**,**D’**) treated with PF-475. (**E**) Percentage of pS935-LRRK2/t-LRRK2 ratio untreated or treated cells with PF-475. One-way ANOVA followed by Tukey’s test. The data are presented as the mean ± SD of three independent experiments (n = 3); * *p*< 0.033.

**Figure 12:**
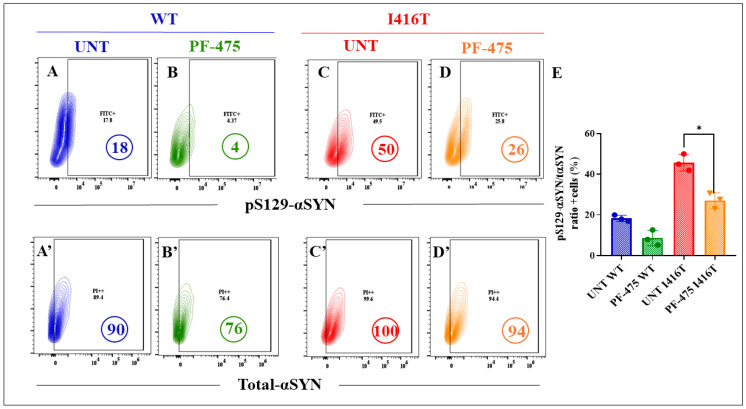
The LRRK2 inhibitor PF-06447475 reduces the levels of phosphorylated protein pS129-α-Syn in PSEN1 I416T DALNs. Representative flow cytometry contour plots show intracellular pS129-αSYN in (**A**,**C**) untreated or (**B**,**D**) treated with PF-475. Representative flow cytometry contour plots show total alpha synuclein protein (t-αSYN) in (**A’**,**C’**) untreated or (**B’**,**D’**) treated cells with PF-475. (**E**) Percentage of pS129-αSYN/t-αSYN ratio untreated or treated cells with PF-475. One-way ANOVA followed by Tukey’s test. The data are presented as the mean ± SD of three independent experiments (n = 3), * *p* < 0.033.

As expected, PF-475 inhibited both basal levels of p-LRRK2 (Figure 11A) by −58% in WT DALNs (Figure 11B) and high endogenous pS935-LRRK2 (Figure 11C) by −60% in mutant DALNs (Figure 11D,E). Notably, PF-475 reduced basal levels of pS129-αSyn (Figure 12A) by −78% in WT DALNs (Figure 12B), whereas it blocked pathological pS129-αSyn (Figure 12C) by −48% in mutant DALNs (Figure 12D,E).

### 2.8. LRRK2 Inhibitor PF-06447475 (PF-475) Decreases the Neuropathologic Apoptotic Markers p-c-JUN and CC3 Associated with FAD and PD in PSEN1 I416T Dopaminergic-like Neurons (DALNs)

**Figure 13:**
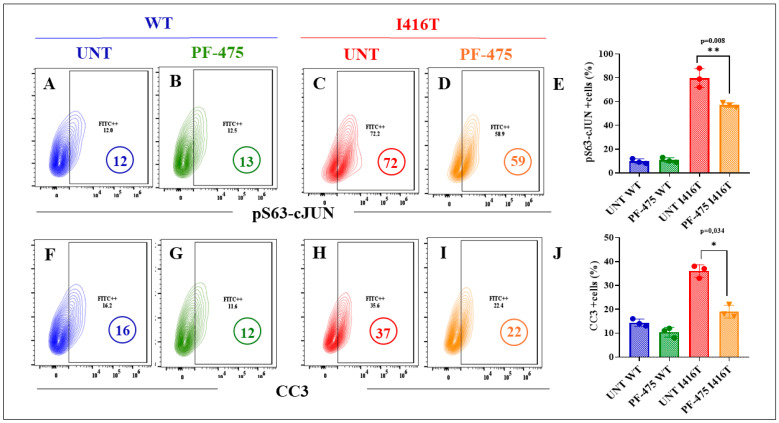
The LRRK2 inhibitor PF-06447475 reduces the levels of c-JUN phosphorylated at serine 63 (pS63-c-JUN) and cleaved caspase-3 (CC3) in PSEN1 I416T DALNs. Representative flow cytometry contour plots show pS63-c-JUN protein level in (**A**,**C**) untreated or (**B**,**D**) treated with PF-475. (**E**) Percentage of pS63-c-JUN expression in untreated or treated cells with PF-475. Representative flow cytometry contour plots show cleaved caspase-3 (CC3) protein in (**F**,**H**) untreated or (**G**,**I**) treated cells with PF-475. (**J**) Percentage of CC3 in untreated or treated cells with PF-475. One-way ANOVA followed by Tukey’s test. The data are presented as the mean ± SD of three independent experiments (n = 3); * *p* < 0.034, and ** *p* < 0.008.

Finally, we evaluated whether PF-475 affected apoptotic signaling in DALNs. As shown in Figure 13, while PF-475 did not affect the levels of pS63-c-Jun in untreated (Figure 13A) or treated WT DALNs (Figure 13B), it decreased the high pS63-c-Jun (Figure 13C) by −18% in mutant DALNs (Figure 13D,E). A similar trend was observed in CC3 when PF-475 was applied to WT and mutant cells. PF-475 did not affect CC3 in WT (Figure 13F,G), but decreased CC3 (Figure 13H) by −41% in PSEN1 I416T DALNs (Figure 13I,J).

## 3. Discussion

We report for the first time that MenSCs carrying the PSEN1 I416T mutation transdifferentiate into DALNs by 70% TH/DAT-positive neurons when cultured in NeuroForsk 2.0 medium for 7 days. This yield of mutant DALNs is comparable to that obtained in WT PSEN1 DALNs as previously reported [35]. Interestingly, the yield of MenSCs-derived DALNs obtained at day 7 and further cultured for 4 days in minimal culture medium (MCm) increased to 81% (+14%) for WT and 96% (+37%) for PSEN1 I416T neurons. This observation suggests that not only can MenSCs transdifferentiate into the DAergic lineage when provided with an appropriate medium (e.g., *NeuroForsk 2.0* medium) for at least 7 days, but the transdifferentiation process can continue in a medium without specific inducers (e.g., forskolin, a cAMP-elevating agent [40] that induces neural-like differentiation of MSCs [41]). This suggests that WT PSEN1 and PSEN1 I416T are molecularly primed for dopaminergic neuronal transdifferentiation. However, further studies are needed to determine the impact of PSEN1 I416T on neuronal differentiation and development [42].

By definition, mixed pathology is a term used to describe a brain tissue, surgical specimen, or postmortem brain that has a mixture of protein alterations or other pathologies [43]. We report that in vitro PSEN1 I416T MenSCs-derived DALNs exhibit typical features of pathological proteins in AD (e.g., Aβ42, pS202/T205 TAU) and PD markers (e.g., pS129-α-Syn); therefore, we propose to expand the definition of mixed pathology to include in vitro cells [23]. Indeed, we found that PSEN1 I1416T DALNs showed accumulation of iAβ, pS202/T205 TAU, and accumulation of pS129-α-Syn, typical pathological diagnostic markers for AD and PD, respectively. This observation suggests that the three proteins may synergistically induce DAergic neuronal degeneration. However, exactly how the iAβ, Tau, and α-Syn proteins interact to converge in PSEN1 I416T DALNs is still unknown. To complicate matters, other proteins such as DJ-1 and LRRK2, appear to play important pathogenic roles in DALNs. Here, we found several lines of evidence suggesting a cascade of molecular events that may explain their putative pathogenic interactions. Consistent with a previous investigation showing that PSEN1 I416T induces the accumulation of iAβ in ChLNs [20], we report that the same mutation induces a high accumulation of iAβ (e.g., +267% compared to WT cells) in DALNs. Therefore, it is reasonable to assume that PSEN1 I416T (and E280A) affects not only the basal forebrain (e.g., NbM (Ch4) neurons, Ref. [44]), hippocampus, and cerebral cortex, but also the midbrain (e.g., substantia nigra). Although several neuropathologic studies have supported such a suggestion for PSEN1 E280A [45,46,47], further neuropathologic postmortem studies in PSEN1 I416T brains are needed to determine the extent of PSEN1 I416T-Aβ-induced presence in the aforementioned cerebral regions. Regardless of which brain region is affected by the I416T mutation, the precise mechanism by which PSEN1 I416T and other mutations produce abnormal levels of iAβ/eAβ is not fully understood [18,48]. Several lines of evidence suggest that the majority of PSEN1 mutations, including E280A [49] and I416T, appear to have a dominant negative effect of the γ-secretase loss-of-function mutants on WT PSEN1 [50,51,52]. Whatever the mechanism, our observations suggest that this mutation increases the accumulation of iAβ in DANLs (and ChLNs, [20]). To date, the exact molecular mechanism(s) of Aβ-induced toxicity have not been definitively determined (e.g., Refs. [53,54,55]). However, the prevailing hypothesis suggests that intracellular Aβ may play a critical role in neuronal toxicity [56,57]. Accordingly, accumulating evidence suggests that iAβ may induce OS either through intramolecular interactions with Aβ (e.g., Met-35 of the Aβ fragment) [58,59,60] or by interacting with mitochondrial organelles [61,62,63], specifically affecting mitochondrial electron transport chain elements such as mitochondrial complexes I, III, and IV [64,65]. Regardless of the mechanism, iAβ directly or indirectly generates ROS, primarily H_2_O_2_ [66]. We found high levels of oxDJ-1, which is associated with iAβ, in mutant DALNs. Since H_2_O_2_ specifically oxidizes the residue C106-SOH (thiol group) to DJ-1C106-SO_3_ (sulfonic group) in protein DJ-1 [67], our observations suggest that PSEN1 I416T induces oxDJ-1 via iAβ > H_2_O_2_. Interestingly, oxidized DJ-1 has been postulated as a postmortem marker of OS in PD brains [68,69]. However, whether oxDJ-1 is present in AD/FAD (E280A, I416T) postmortem brains has not been systematically investigated. Therefore, further neuropathological analyses are needed to confirm or exclude the co-occurrence of oxDJ-1 and Aβ in pathological human brains.

Interestingly, we found a high abnormal percentage of pS395 LRRK2 in PSEN1 I416T DALNs. This observation may be explained by the fact that, in addition to oxidizing DJ-1, H_2_O_2_ could activate LRRK2 kinase activity by directly enhancing its autophosphorylation, e.g., at T1967 [70], S2032, and T2035 [71,72,73], or indirectly via phosphorylation of S910 and S935 by the inhibitor of nuclear factor-κB (IκB) kinase (IKK) complex [74]. Once active, LRRK2 kinase has been shown to trigger the activation of at least six key proteins associated with protein aggregation and cell death: (i) LRRK2 directly interacts with dynamin-like protein 1 (DLP1), a key mitochondrial fission protein, increasing its mitochondrial targeting and thereby promoting mitochondrial fragmentation [75]; (ii) increases the phosphorylation of peroxiredoxin 3 (PRXD3), exacerbating OS-induced cell death [76]; (iii) phosphorylates both activation of apoptosis signal-regulating kinase 1 (ASK1) [77] and MKK4/MAPK kinase [78], thereby triggering the JNK/c-JUN/PUMA death pathway; and (iv) phosphorylates the pro-apoptotic transcription factor TP53 [79], thereby triggering downstream apoptotic signaling. In addition, (v) LRRK2 kinase phosphorylates α-Syn at S129 [24], which is the major component of pathological deposits in PD [80], and (vi) p-LRRK2-induced JNK induces p-TAU at S202/T205 [81]. Consistent with these observations, we found that pS935-LRRK2 was associated with an abnormal increase in pS63-c-JUN, PUMA, TP53, pS129 α-Syn, and pS202/T205 TAU-positive cells in PSEN1 I416T DALNs. These observations suggest that LRRK2 kinase converge in phosphorylating α-Syn and activating TP53. Taken together, these observations suggest that LRRK2 may function as proapoptotic kinase under OS stimuli. Consistent with others [82], JNK activation is driven by iAβ and is associated with TAU pathology in DALNs. Our findings may explain why parkinsonism associated with pure AD is associated with significant neuronal loss in both the SN and putamen, suggesting alterations in the nigrostriatal pathway [83]. Furthermore, abundant Aβ (100%) pathology as well as α-Syn (63.6%) and pS202/T205 TAU (100%) were found in G2019S LRRK2 mutant carriers (in which kinase activity is exacerbated) in the midbrain [84].

Apoptosis is a prominent feature of neuronal cell death in both AD [85] and PD [86]. Therefore, the identification of key proteins involved in this cell death is essential for therapeutic purposes. Here, we show that DALNs endogenously exhibit activation of the apoptogenic transcription factors c-JUN and TP53, most likely through phosphorylation of LRRK2 kinase, as mentioned above in mutant DALNs. Interestingly, both c-JUN and TP53 have been shown to transcriptionally activate PUMA [87,88,89], a BH3-only protein that functions as an important regulator of induced apoptosis [90]. Unfortunately, no data are available in postmortem FAD midbrain to validate our present findings. Nevertheless, PUMA, together with other proapoptotic proteins (e.g., BAX/BAK), appears to promote mitochondrial outer membrane permeabilization (MOMP), thereby releasing mitochondrial apoptotic proteins such as cytochrome c, which, in turn, indirectly activates the executioner protein caspase 3 (CASP3) to cleaved caspase 3 (CC3). This protease not only degrades the nucleus [91], but also disrupts the cytoarchitecture of neurons [92], among other target proteins (e.g., poly(ADP-ribose) polymerase, gelsolin, and DNA-dependent kinase). Therefore, the labeling of CC3-positive cells is an apoptotic marker [93]. We found that under the present experimental conditions, PSEN1 I416T induced an intrinsically high percentage (~40%) of CC3-positive markers in DALNs. Taken together, our results suggest that PSEN1 I416T DALNs are susceptible to cell death by apoptosis.

Previously, PSEN1 I416T ChLNs were shown to be unresponsive to ACh-induced Ca^2+^ influx [20], likely through eAβ42 specific binding to alpha7 nicotinic acetylcholine receptors (α7nAChRs, [94,95,96]). Similarly, we report for the first time that PSEN1 I416T DALNs are unresponsive to DA-induced Ca^2+^ influx, likely through eAβ42 specific binding to either dopamine autoreceptors [97] or voltage-gated Ca^2+^ channels [98]. Most interestingly, transdifferentiated WT DALNs responded to DA stimuli by exhibiting two maximal fluorescence changes (ΔF/F) of transient Ca^2+^ influx at 10 and 100 s. This observation suggests that DALNs are functional. However, further investigation is needed to determine the identity of such receptors and their possible interactions with eAβ42. Furthermore, although several studies have shown that the DAergic system is severely affected in AD [99], the mechanism underlying its effects in the pathogenesis of AD remains unclear. Therefore, our observations provide an in vitro FAD model to explore natural or synthetic agents to reverse the DA-induced Ca^2+^ dysfunctional response in DALNs.

Several data suggest that LRRK2 functions as a master protein kinase involved in the regulation of cellular apoptosis. Indeed, genetic ablation or pharmacological inhibition of LRRK2 protects nerve-like cells [29,33], HEK-293 cells [35], *Drosophila melanogaster* [27,100], human microglial cells [101], and rats [34] against rotenone, paraquat, trichloroethylene, tetrachloroethylene, manganese, and maneb-induced OS, apoptosis, and locomotor impairment as a model of PD. In this study, we demonstrate that pharmacological inhibition of LRRK2 reversed PSEN1 I416T-induced proteinopathy (iAβ, p-TAU, α-Syn), OS (oxDJ-1), and apoptosis (p-c-JUN, TP53, PUMA, CC3) in DALNs. Our data suggest that PSEN1 I416T induces a mechanism that abnormally phosphorylates TAU protein in a manner similar to that seen in cells carrying the LRRK2 G2019S mutation, a mutation that keeps LRRK2 kinase active through H_2_O_2_ [102]. Overall, LRRK2 kinase inhibition reversed the PSEN1 I416T mutation-dependent effects on the progression of DALNs pathology.

## 4. Materials and Methods

### 4.1. Transdifferentiation of Menstrual Blood-Derived Mesenchymal Stem Cells (MenSCs) into Dopaminergic-like Neuron (DALN) Cells

The differentiation protocol was performed according to reference [35]. Menstrual blood samples were collected from a healthy woman (Tissue Bank Code, TBC #72209) and an asymptomatic FAD patient (TBC #45000). Menstrual sample donors provided a signed informed consent approved by the Ethics Committee of the Sede de Investigación Universitaria (SIU), University of Antioquia, Medellín, Colombia (Act 20-10-846). Informed consent was obtained from all subjects included in the study. Regular culture medium (RCm) containing low-glucose DMEM (Sigma-Aldrich cat#D5546, St. Louis, MO, USA) and 10% FBS ((Sigma-Aldrich cat#12103C, St. Louis, MO, USA) was used to seed MenSCs on laminin-coated culture plates at a density of 1–1.5 × 10^4^ cells/cm^2^ for 24 h. Subsequently, the medium was replaced with dopaminergic differentiation medium (also known as *NeuroForsk 2.0* medium) containing Neurobasal TM medium (Gibco cat# 21103-049, Grand Island, NY, USA), 2% B-27 supplement (Gibco cat# 12587-010, Grand Island, NY, USA), and a cocktail of nerve growth factors as published elsewhere [35] at 37 ◦C for 7 days. The PSEN1 I416T DALN cells (obtained after seven days in *NeuroForsk 2.0*) were further cultured in minimal culture medium (MCm) containing low-glucose DMEM and 1% FBS for an additional four days after transdifferentiation, as *NeuroForsk 2.0* contains a number of components that could potentially interfere with the interpretation and measurements of the experiment.

### 4.2. Flow Cytometry Analysis

#### 4.2.1. Detection of Dopaminergic Linage Markers

Flow cytometry was used to determine the percentage of NFL and DAT/TH double- positive cells based on previous reports. Briefly, WT or PSEN1 I416T DALNs at 7 and 11 days of differentiation were detached with 0.25% trypsin (Sigma-Aldrich, cat# T4799 Sigma-Aldrich; St. Louis, MO, USA) and washed 1× in PBS. Cells (1 × 10^5^) were then resuspended in PBS, fixed in cold 80% ethanol, and stored at −20 °C. The cells were then washed with PBS and permeabilized with 0.2% Triton X-100 (Cat# 93443, Sigma-Aldrich; St. Louis, MO, USA) plus 1.5% bovine serum albumin (BSA, Cat# A9418, Sigma-Aldrich; St. Louis, MO, USA) in phosphate-buffered solution (PBS) for 30 min. The cells were then washed and incubated with primary antibodies (1:200; diluted 1× in PBS containing 0.1% BSA) against tyrosine hydroxylase protein (TH cat# abx019187 abbexa, Cambridge, UK), dopamine transporter (DAT, cat# abx104987 abbexa, Cambridge, UK).

#### 4.2.2. Detection of Alzheimer’s and Parkinson’s Neuropathological, Protein Kinases, Oxidative Stress, and Cell Death Markers

For the analysis of Alzheimer and Parkinson disease markers-, oxidative stress- and cell death-related markers, the WT or PSEN1 I416T DALNs at 11 days of differentiation cultured with MCm without or with PF-475 (1 µM) were detached with trypsin 0.25% (Sigma Aldrich, cat# T4799 Sigma-Aldrich; St. Louis, MO, USA) and washed in PBS 1×. After that, cells (1 × 10^5^) were resuspended in PBS and fixated with cold 80% ethanol and stored at 20 °C. Then, cells were washed with PBS and permeabilized with 0.2% triton X-100 (Cat# 93443, Sigma-Aldrich; St. Louis, MO, USA) plus 1.5% bovine serum albumin (BSA, Cat# A9418, Sigma-Aldrich; St. Louis, MO, USA) in phosphate-buffered solution (PBS) for 30 min. Then, cells were washed and incubated with primary antibodies (1:200; diluted in PBS 1× containing 0.1% BSA) against APP751 and/or protein amyloid β1–42 (1:500; clone 6E10 cat# 803014, Biolegend, San Diego, CA, USA), oxidized DJ-1 (ox(Cys^106^)DJ-1; spanning residue Cys^106^ of human PARK7/DJ1; oxidized to produce cysteine sulfonic (SO_3_) acid; (cat # ab169520, Abcam; Boston, MA,USA), total TAU (1: 500; t-Tau; cat# T6402, Sigma-Aldrich; St. Louis, MO, USA), phospho-TAU (p-Tau, 1:500, Ser^202^/Thr^205^, cat# MN1020 (AT8), Thermo Fisher Scientific; Whaltam, MA, USA), α-synuclein (Ser^129^; cat# AB51253 Abcam; Boston, MA, USA), antibodies, total LRRK2 (1:200 cat # MA5-11154, Thermo Fisher Scientific; Whaltam, MA, USA), phospho-LRRK2 (p-LRRK2, 1:200 Ser^935^ cat# ab133450, Abcam; Boston, MA, USA), antibodies. To assess cell death, we used primary antibodies against p53-upregulated modulator of apoptosis (1:500; PUMA, cat# ab-9643, Abcam; Boston, MA, USA), p53 (1:500; cat# MA5-12-453, Millipore, Merck, Darmstadt, DE), phospho-c-Jun (1:250; c-Jun (S63/73) cat#sc-16312, Santa Cruz; Dallas, TX, USA), and caspase-3 (1:250; cat # AB3623, Millipore, Merck, Darmstadt, Germany). This was followed by incubation with secondary fluorescent antibodies according to Ref. [35]. Resuspended cells were analyzed using BD LSR Fortessa II flow cytometer (BD Biosciences, Franklin Lakes, NJ, USA). Ten thousand events were acquired, and the acquisition analysis was performed using FlowJo 7.6.2 (https://www.flowjo.com/previous-versions-flowjo, accessed on 15 January 2025) data analysis software.

### 4.3. Immunofluorescence Analysis

For immunofluorescence analysis, WT or PSEN1 I416T DALNs at 11 days of differentiation cultured with MCm without or with PF-475 (1 µM) were fixed with paraformaldehyde for 20 min, followed by Triton X-100 (0.1%) permeabilization and 5% bovine serum albumin (BSA) blocking. The cells were then incubated with the above primary antibodies (1:200) overnight. After thorough rinsing, the cells were incubated with secondary fluorescent antibodies (DyLight 488 and 595 donkey anti-rabbit, goat, and mouse, Cat DI 2488 and DI 1094, respectively, 1:500). The nuclei were stained with Hoechst 33,342 (1 M, Life Technologies, Carlsbad, CA, USA), and images were captured on a Zeiss Axiostart 50 fluorescence microscope equipped with a Zeiss AxioCam Cm1 (Zeiss Wöhlk-Contact-Linsfluoreen, Gmbh, Schökirchen, Germany), and image processing was performed according to the online guidance program (http://imagej.nih.gov/ij/, accessed on 15 February 2025) [103]. Mean fluorescence intensity (MFI) was calculated using ImageJ (http://imagej.nih.gov/ij/, accessed 15 February 2025) according to the formula: mean fluorescence intensity (background corrected) = mean gray value (ROI)—mean gray value (background).

### 4.4. Intracellular Calcium Imaging

Cytoplasmic Ca^2+^ concentration ([Ca^2+^]_i_ [104]) was measured according to ref. [105]. Briefly, WT or PSEN1 I416T DALNs at 7 and 11 days of differentiation were transferred to a bath solution (NBS; in mM: 137 NaCl, 5 KCl, 2.5 CaCl_2_, 1 MgCl_2_, 10 HEPES, pH 7.3, and 22 glucose) containing a Ca^2+^-sensitive indicator (2 µM Fluo-3-AM, an acetoxymethyl ester form of the fluorescent dye Fluo-3; Thermo Fisher Scientific Cat# F1242, Whaltam, MA, USA) for 30 min at RT and then washed five times. Intracellular Ca^2+^ transients were elicited by dopamine (DA, 1 mM final). Fluorescence microscopy images were acquired using a Zeiss Axiostart 50 fluorescence microscope equipped with a Zeiss AxioCam Cm1 (Zeiss Wöhlk-Contact-Linsfluoreen, Gmbh, Schoenkirchen, Germany). Prior to imaging, several regions of interest (ROIs) were defined in the field of view of the camera. One of the ROIs was cell-free, and the fluorescence intensity measured here was considered as “background fluorescence” (F_bg_). In the “kinetic view” mode, the program calculated and displayed the average fluorescence intensities of the ROIs in arbitrary units (AU) as a function of time. In the “image view” mode, the time dependence of the spatial distribution of the fluorescence emission was displayed, and the fluorescence intensities (and, thus, the Ca^2+^ levels) were represented by pseudocolors. To calculate the changes in average Ca^2+^-dependent fluorescence intensities in the “kinetic view” mode, first the F_bg_ value was determined from the cell-free ROI, then the resting fluorescence intensities (F_rest_) of the cell-containing ROIs were obtained as the average of the points recorded during a period of 10 s before the addition of DA. Peaks of fluorescence transients were found by calculating the average of three consecutive points and identifying the points that gave the highest average value (F_max_). The amplitudes of the Ca^2+^-induced fluorescence transients were expressed relative to the resting fluorescence (ΔF/F) and calculated using the formula ΔF/F = (F_max_ − F_rest_)/(F_rest_ − F_bg_). Image data processing was performed according to the online guidance program (http://imagej.nih.gov/ij/) [103].

### 4.5. Data Analysis

In this experimental design, a vial of either wild-type or PSEN1 I416T MenSCs was thawed and cultured, and the cell suspension was pipetted into different wells of a 24-well plate at a standardized cell density of 2 × 10^4^ cells per cm^2^. Cells (i.e., biological and observation units) were randomized to wells by simple randomization (sampling without replacement method), and then wells (i.e., experimental units) were randomized to treatments by a similar method. Sample size estimation was performed according to the guidelines proposed by [106], considering the expected effect size, variance, and desired statistical power to ensure adequate sensitivity to detect significant differences. Experiments were conducted in triplicate. Data from individual replicate wells were averaged to give a value of n = 1 for that experiment, and this was repeated three times blinded to the experimenter and/or flow cytometer analyst for a final value of n = 3. This experimental design was performed as previously described in [20] and based on the statistical considerations described in ref. [106]. Provided that the data from the experimental unit (i.e., the well) satisfy the independence of observations, the dependent variable is normally distributed in each treatment group (Shapiro–Wilk test), and the variances are homogeneous (Levene’s test). After confirming normality and homoscedasticity assumptions, statistical significance was determined by one-way or two-way ANOVA followed by Tukey’s post hoc comparison with a 95% confidence interval using GraphPad Prism version 10.0 software. Differences between groups were considered significant only when the *p*-value was 0.05 (*), 0.01 (**), and 0.001 (***). All data are expressed as mean ± S.D.

## 5. Conclusions

**Figure 14:**
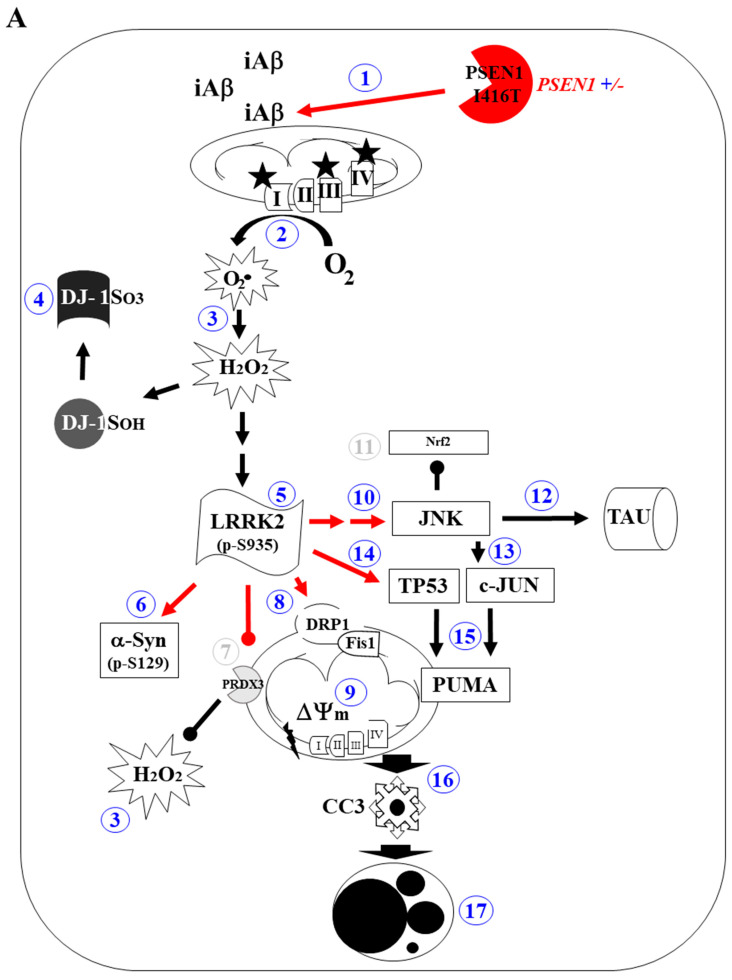

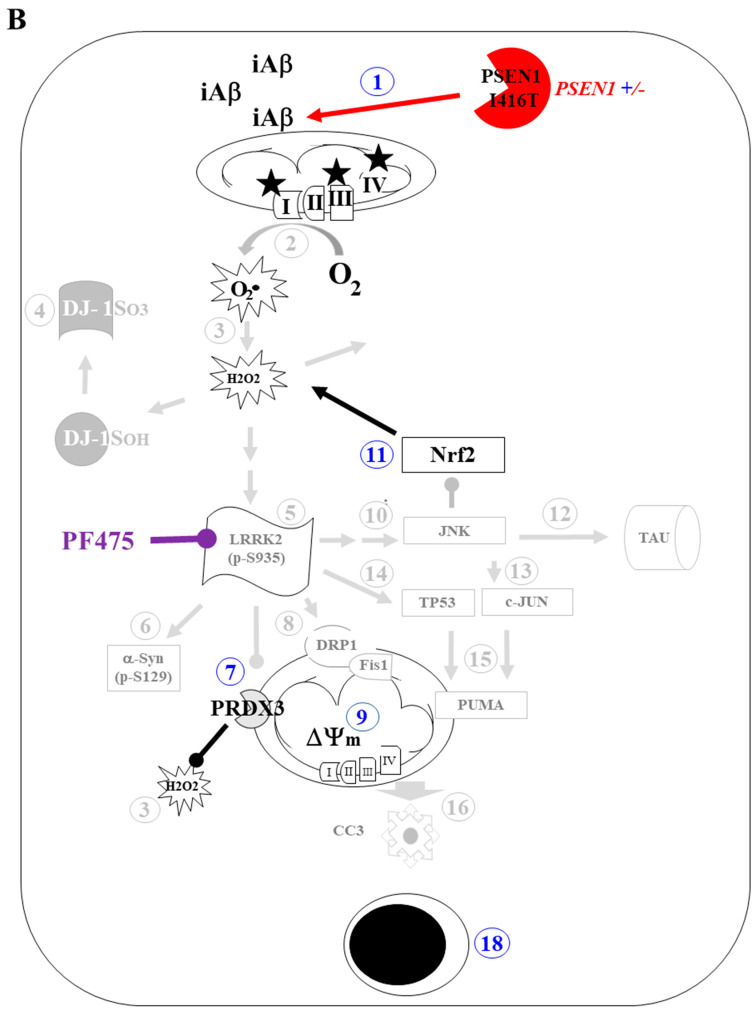
Schematic model of cell signaling induced by PSEN1/γ-secretase and effect of LRRK2 inhibitor PF475 in PSEN1 I416T DALNs. (**A**) PSEN1/γ-secretase signaling mechanism. The PSEN1+/- gene encodes a 467 aa catalytic and non-catalytic PSEN1 I146T protein that alters the metabolism of the type I transmembrane protein APP by a mechanism that is not fully understood, resulting in the overproduction of iAβ (1). The iAβ peptide acts on mitochondrial complex I (NADH: ubiquinone oxidoreductase), complex III (Q-cytochrome c oxidoreductase), or complex IV (cytochrome c oxidase) (2), interfering with the electron transport chain and simultaneously generating the anion superoxide (O2^.-^) and hydrogen peroxide (H_2_O_2_, 3). The latter is not only capable of oxidizing the stress sensor protein DJ-1Cys106-SH to DJ-1Cys106-SO_3_ (4), but also directly activates leucine-rich repeat kinase 2 (LRRK2) kinase directly through autophosphorylation or indirectly through kinase (e.g., via IKK) signaling (5). Once LRRK2 is phosphorylated at S935, the active pS935-LRRK2 kinase activates at least six major targets: (i) alpha-synuclein (αSyn) at the pathological residue S129 (6); (ii) inactivates protein peroxiredoxin 3 (PRDX3, 7), preventing H_2_O_2_ catalysis; (iii) activates the mitochondrial fission protein DLP-1 (dynamin-like protein 1, 8), which together with the fission protein-1 (Fis-1) receptor induces loss of mitochondrial potential (ΔΨm, 9); (iv) pS935-LRRK2 indirectly phosphorylates JNK (10), which, in turn, represses nuclear factor erythroid 2-related factor 2 (Nrf2)-associated expression of antioxidant proteins (11), phosphorylates the protein TAU (12), and activates c-JUN (13); (v) activates the transcription factors TP53 (14). Both pS63-c-Jun and TP53 factors transcribe PUMA (15), a Bcl-2-only protein involved in further mitochondrial depolarization (9). Impairment of mitochondrial potential leads to the release of apoptogenic proteins (e.g., cytochrome C), resulting in the production of cleaved caspase 3 (16), which is responsible for chromatin condensation and DNA fragmentation (17), a typical apoptotic feature in PSEN1 I416T DALNs. (**B**) Upon exposure to PF475, p-LRRK2-associated apoptosis signaling is drastically reduced (faint black line). As a result, PRDX3 activity is restored (7), Nfr2-induced antioxidant protein expression is increased (11), Aβ-induced ROS/H_2_O_2_ is decreased (2,3), and ΔΨm is increased (9). These actions result in global neuronal recovery and survival of PSEN1 I416T DALNs (18).

We demonstrate for the first time that PSEN1 I416T MenSCs transdifferentiate into DALNs in *NeuroForsk 2.0* medium and that the mutation stimulates a high yield of DALNs. The I416T mutation significantly induced the accumulation of iAβ/(H_2_O_2_), which, in turn, induced the activation of LRRK2 kinase. Its activation leads to the accumulation of α-Syn, p-TAU, and cell death signaling. A proposed mechanism by which PSEN1 I146T induces proteinopathy, OS, and apoptosis in DALNs is shown in Figure 14A. Interestingly, the LRRK2 kinase inhibitor PF-475 reverses the PSEN1 I146T-induced mixed pathology markers in DALNs (Figure 14B). This investigation demonstrates the coexistence of Alzheimer’s pathology markers (i.e., iAβ, pS202/T205 TAU) with Parkinson’s pathology markers (i.e., pS129-α-Syn) in PSEN1 I416T DALNs. We conclude that the observed neuroprotective effects of PF-475 are due to direct inhibition of LRRK2 activity. In addition, we speculate that PF-475 indirectly enhances the autophagy–lysosome pathway [107], thereby significantly reducing the intracellular accumulation of iAβ. However, further studies are needed to clarify this issue. Inhibition of LRRK2 with tyrosine kinase inhibitors (e.g., PF-475) may be a means to prevent OS and apoptosis in PSEN1 I416T DALNs.

## Data Availability

All datasets generated for this study are included in the manuscript.
